# Swine Leukocyte Antigen Diversity in Canadian Specific Pathogen-Free Yorkshire and Landrace Pigs

**DOI:** 10.3389/fimmu.2017.00282

**Published:** 2017-03-15

**Authors:** Caixia Gao, Jinqiang Quan, Xinjie Jiang, Changwen Li, Xiaoye Lu, Hongyan Chen

**Affiliations:** ^1^Laboratory Animal and Comparative Medicine Team, State Key Laboratory of Veterinary Biotechnology, Harbin Veterinary Research Institute, Chinese Academy of Agricultural Sciences (CAAS), Harbin, China

**Keywords:** major histocompatibility complex, swine leukocyte antigen, diversity, specific pathogen-free, Yorkshire, Landrace

## Abstract

The highly polymorphic swine major histocompatibility complex (MHC), termed swine leukocyte antigen (SLA), is associated with different levels of immunologic responses to infectious diseases, vaccines, and transplantation. Pig breeds with known SLA haplotypes are important genetic resources for biomedical research. Canadian Yorkshire and Landrace pigs represent the current specific pathogen-free (SPF) breeding stock maintained in the isolation environment at the Harbin Veterinary Research Institute, Chinese Academy of Agricultural Sciences. In this study, we identified 61 alleles at five polymorphic SLA loci (*SLA-1, SLA-2, SLA-3, DRB1*, and *DQB1*) representing 17 class I haplotypes and 11 class II haplotypes using reverse transcription-polymerase chain reaction (RT-PCR) sequence-based typing and PCR-sequence specific primers methods in 367 Canadian SPF Yorkshire and Landrace pigs. The official designation of the alleles has been assigned by the SLA Nomenclature Committee of the International Society for Animal Genetics and released in updated Immuno Polymorphism Database-MHC SLA sequence database [Release 2.0.0.3 (2016-11-03)]. The submissions confirmed some unassigned alleles and standardized nomenclatures of many previously unconfirmed alleles in the GenBank database. Three class I haplotypes, Hp-37.0, 63.0, and 73.0, appeared to be novel and have not previously been reported in other pig populations. One crossover within the class I region and two between class I and class II regions were observed, resulting in three new recombinant haplotypes. The presence of the duplicated *SLA-1* locus was confirmed in three class I haplotypes Hp-28.0, Hp-35.0, and Hp-63.0. Furthermore, we also analyzed the functional diversities of 19 identified frequent SLA class I molecules in this study and confirmed the existence of four supertypes using the *MHCcluster* method. These results will be useful for studying the adaptive immune response and immunological phenotypic differences in pigs, screening potential T-cell epitopes, and further developing the more effective vaccines.

## Introduction

Swine major histocompatibility complex (MHC), which codes for swine leukocyte antigen (SLA), has been mapped to pig chromosome 7 spanning the centromere. SLA genes are highly polymorphic comprising classical class I, II, and III genes (1). SLA class I and class II proteins are involved in the adaptive immune response through their respective presentations of endogenous and exogenous peptide antigens to circulating T lymphocytes (2). Class I molecules are recognized by cytotoxic T lymphocytes, which kill transformed cells and virally infected cells, thus preventing tumor cell replication or pathogen release. Class II molecules are recognized by helper T cells, which induce or permit immune responses, including the production of antibodies to extracellular pathogens. Class III molecules include many important immune-defense genes, such as the tumor necrosis factor gene families and components of the complement cascade (3). Among SLA class I and class II genes, *SLA-1, SLA-2, SLA-3, DRB1*, and *DQB1* are highly polymorphic. Up to now, 192 classical SLA class I alleles (69 *SLA-1*, 87 *SLA-2*, and 36 *SLA-3*) and 145 polymorphic class II alleles (91 *DRB1* and 54 *DQB1*) have been designated by the SLA Nomenclature Committee of the International Society for Animal Genetics (ISAG) in the Immuno Polymorphism Database (IPD)-MHC SLA sequence database.^1^

Yorkshire and Landrace pigs are widely distributed in the world. When every country develops breeding according to their need, characteristics of natural populations are kept, and the certain special features are bred. These two breeds are the famous leading lean hogs around the world. There are few reports using them as experimental animals for veterinary and medical studies. However, pigs have an evolutionary resemblance to humans and share anatomical, physiological, immunological, metabolic, and nutritional similarities, making them promising organ donors for xenotransplantation. Additionally, because of their white fur, small genetic differentiation, stable genetic structure, and lack of major swine diseases as well as the corresponding antibodies, Canadian Yorkshire and Landrace pigs are more suitable as animal models for the study of virus infections and pathogenesis, host immunological responses, vaccine evaluation, and the identification of T-cell epitopes. However, the different allelic forms of the SLA complex have been confirmed to bind different classes of peptides, and the presence of unknown SLA types in pigs could thus potentially confound the results of such studies (4–6). SLA typing is therefore necessary to our understanding of SLA peptide antigen binding and presentation. Determining which SLA alleles are expressed by the animals is an essential initial step in identifying virally derived T-cell epitopes that play a role in generating protective T cell responses against infection in pigs. Following SLA typing, candidate peptide antigens can be screened by various techniques, such as cytotoxic lymphocyte peptide and cytokine stimulation, peptide MHC affinity and stability assays, flow cytometric analyses of T-cell populations specific for peptide MHC tetramers, and by the web resource ICES index^2^ (7–12). In this process, the identification of peptides binding to SLA alleles is a critical step. The *MHCcluster* method just provides an important tool for functionally clustering MHC molecules based on their predicted binding specificities (13).

In this study, we characterized the alleles of five polymorphic SLA loci (*SLA-1, SLA-2, SLA-3, DRB1*, and *DQB1*) identified in Canadian specific pathogen-free (SPF) Yorkshire and Landrace parental breeding pigs using sequence-based typing (SBT), followed by use of a rapid polymerase chain reaction sequence-specific primer (PCR-SSP) method for SLA typing and to assign alleles to haplotypes in the progeny. Following identification of the common SLA class I alleles in Yorkshire and Landrace pigs, we then used the *MHCcluster* method to compare the peptide-binding functions of their encoded proteins. The results of this study will help to develop Yorkshire and Landrace pigs with well-defined SLA as important and useful laboratory animals for investigating pathogenic and immunological mechanisms for various porcine pathogens, as well as for furthering our understanding of the structural and functional implications of SLA gene polymorphisms.

## Materials and Methods

### Animals

Yorkshire and Landrace pigs in this study were imported from Canadian Genesus incorporated in 2014 and represent the current breeding stock maintained in the isolation environment at the Harbin Veterinary Research Institute of the Chinese Academy of Agricultural Sciences. All the pigs have been free of 12 major swine diseases as well as their corresponding antibodies, including foot and mouth disease, classical swine fever, porcine reproductive and respiratory syndrome, Japanese encephalitis, pseudorabies, transmissible gastroenteritis, *Brucella, Bordetella bronchiseptica, Actinobacillus pleuropneumoniae*, swine mycoplasmal pneumonia, swine influenza, and porcine epizootic diarrhea. Therefore, they are excellently suited as laboratory animals for biomedical research. In this study, blood samples were obtained from 37 parental breeding pigs, including 15 Yorkshire (4♂, 11♀) and 22 Landrace (6♂, 16♀) pigs, for isolation of peripheral blood mononuclear cells. Ear tissues were collected from 330 F1 progeny pigs for DNA extraction, including 113 Yorkshire, 157 Landrace, and 60 crossbred Yorkshire and Landrace progeny pigs. The swine pedigree was showed in Table S1 in Supplementary Material. All sample collections were approved by the ethical review board of Harbin Veterinary Research Institute of the Chinese Academy of Agricultural Sciences and performed in accordance with animal ethics guidelines and approved protocols. The animal Ethics Committee approval number is Heilongjiang-SYXK-2006-032.

### Identification of SLA Alleles by SBT

Five pairs of full-length locus-specific primers were used to amplify the complete coding sequences of five SLA loci (*SLA-1, SLA-2, SLA-3, DRB1*, and *DQB1*). The primers for *SLA-2, SLA-3, DRB1*, and *DQB1* genes were as described by Ho et al. (14). The primers for the *SLA-1* locus were redesigned as follows: SLA1-F (5′-CTC AGC TTC TCC CCA GAC CCC GAG GCT GAG GAT C-3′) and SLA1-R (5′-GGA TTC TGG AAG GTT CTC AAT CCT TCC ATT TAT TTC CTC-3′). Extraction of total RNA from peripheral blood mononuclear cells, synthesis of cDNA, and reverse transcription-PCR amplification were carried out as described previously (15). All PCR products were purified, and nucleotide sequences were determined by direct and cloning sequencing. For direct sequencing, two internal sequencing primers, SLA1f (5′-GGC TTC TAC CCT AAG GAG A-3′) and SLA1r (5′-GCC CAC TTC TGG AAG GT-3′), were used to determine the cDNA sequences of three class I genes (*SLA-1, SLA-2*, and *SLA-3*). All sequences were submitted to the GenBank database. Compared with the published SLA alleles in the IPD-MHC SLA sequence database, novel alleles as well as the corresponding haplotypes were assigned official names by the ISAG SLA Nomenclature Committee.

### SLA Genotyping by PCR-SSP

A total of 330 F1 progeny of Canadian SPF Yorkshire and Landrace pigs were genotyped for their SLA class I (*SLA-1, SLA-2*, and *SLA-3*) and class II (*DRB1* and *DQB1*) alleles by PCR-SSP method using extracted DNA samples. These included 113 Yorkshire, 157 Landrace, and 60 crossbred Yorkshire and Landrace progeny. All animals were SLA-typed using the complete set of primers specific for the alleles at all five SLA loci and most of primers followed that by Ho et al. (16, 17) (Table S2 in Supplementary Material). The reaction volumes, cycling conditions, and interpretation of PCR amplification were performed as described previously (15). The *ACTA1* gene (porcine α-actin) was used as a positive control, and a negative control without DNA was setup for each typing, to check for reagent contamination (lane 1).

### *MHCcluster* Analysis

Comparative analysis of peptide-binding function was performed using the *MHCcluster* method^3^ for in-house and identified frequent SLA class I alleles in this study. In brief, *MHCcluster* was used to cluster SLA molecules functionally based on their predicted binding specificity to 50,000 random natural 9 amino acid long peptides (13). This analysis predicts binding motifs for SLA class I molecules.

## Results

### SLA Diversity in Yorkshire and Landrace Pigs

A total of 43 class I (16 *SLA-1*, 16 *SLA-2*, and 11 *SLA-3*) and 18 class II alleles (8 *DQB1* and 10 *DRB1*) were identified at five SLA loci in 15 Yorkshire and 22 Landrace pigs by SBT (Table 1; Data Sheet S1 in Supplementary Material). All the nucleotide sequences and protein sequences have been submitted to the GenBank database under the accession numbers KU754544–KU754601 and KU953375–KU953377. Comparisons with other published sequences in the IPD-MHC SLA sequence database indicated that three of the alleles were novel and the official allele names SLA-3*05:03:02, DRB1*06:07, and DQB1*02:12 were assigned by the ISAG SLA Nomenclature Committee. The official designation of alleles identified in this study confirmed some unassigned alleles and standardized nomenclatures of many previously unconfirmed alleles in the GenBank database (Table 2). The nucleotide sequences of four alleles identified in the 16 samples by SBT were identical to those with GenBank accession numbers KX056221, KT194212, and KT350995; AK351685; DQ992497, DQ992498, and DQ992499; and AK239592, respectively, without official allele names. Therefore, the four confirmed allelic sequences were designated as: SLA-1*07:04 (KU754554), SLA-1*08:11 (KU754558), SLA-2*16:03 (KU754568), and SLA-2*10:06 (KU754570), respectively, by the Committee. Furthermore, SLA-1*08pt13, SLA-1*08sk11, SLA-1*11jh02, SLA-2*w09pt22, SLA-2*10es21, SLA-2*10sk21, SLA-3*03pt31, and SLA-3*hb06 alleles had previously been identified in other pig breeds and some cell lines, e.g., Korean native pigs, Danish swine, Landrace pigs, cell lines “ESK-4, PT-K75, SK-RST,” and so on (18–21). These alleles were therefore assigned the following permanent names by the SLA Nomenclature Committee: SLA-1*08:07 (KU953375), SLA-1*08:08 (KU754548), SLA-1*11:03 (KU754550), SLA-2*09:03 (KU953376), SLA-2*10:04 (KU754565), SLA-2*10:05 (KU754566), SLA-3*03:06 (KU953377), and SLA-3*04:04 (KU754583), respectively. The nucleotide sequences of SLA-1*rh03 (AF074427), SLA-2*05rh03 (AF074428), DRB1*06sL47 (L08847), DQB1*0203 (AB012093, AF113970), and DQB1*0204 (L08844, EU039916) had previously been submitted to the IPD-MHC database and were identical to the partial coding sequences (exons 2 and 3 of SLA class I genes and exon 2 of class II genes) that we identified in SPF Yorkshire and Landrace pigs. Therefore, the official allele names were assigned as follows by the committee, according to the SLA nomenclature rules (22–24): SLA-1*07:03 (KU754555), SLA-2*05:05 (KU754569), DRB1*06:07 (KU754601), DQB1*02:03 (KU754591), and DQB1*02:12 (KU754590), respectively. The other official allele designations assigned by the ISAG SLA Nomenclature Committee are shown in Table S2 in Supplementary Material. IPD-MHC database has been updated in 2016 and the confirmed alleles in this study have been released in database [Release 2.0.0.3 (2016-11-03)]. In addition, more than two *SLA-1* alleles each were detected in seven of the 37 SBT-typed pigs, indicating the presence of a duplicated *SLA-1* locus in multiple SLA haplotypes in Canadian SPF Yorkshire and Landrace pigs. Notably, the SLA-1*09:01 allele was not detected by SBT method using the full-length locus-specific primers SLA1-F/SLA1-R in seven Landrace pigs with Hp-28.0. Subsequently, we attempted to use a pair of sequence-specific typing primers to amplify this allele by PCR in nine Landrace parental breeding pigs (pig ID: 2922, 3037, 3093, 3111, 3112, 3219, 3220, 3222, and 3241). We found clear amplified bands of the expected sizes (193 bp) were visualized in seven pigs with Hp-28.0 (except pig 3037 and 3093 with Hp-73.0).

**Table 1:** **Alleles of five swine leukocyte antigen (SLA) loci identified in Canadian specific pathogen-free Yorkshire and Landrace pigs by the sequence-based typing (SBT) method**.

Pig ID	Sex	SLA allele specificity					Corresponding SLA genotype^a^
		*SLA-1*	*SLA-3*	*SLA-2*	*DRB1*	*DQB1*	
**Landrace**							
2906	♀	07:02, 14:01	05:02, 04:02	02:02, 10:04	06:01, 10:01	06:01, 07:01:01	Hp-32.12b/64.23
2922	♀	14:01, 15:01, 09:01^b^	05:02, 07:01:02	10:04, 05:03	10:01	06:01	Hp-64.23/28.23
2959	♀	14:01, 08:11	05:02	10:04, 10:06	10:01	06:01	Hp-64.23/26b.23
2995	♀	14:01	05:02, 04:04	10:04, 06:02:01	10:01, 06:02	06:01, 07:01:01	Hp-64.23/62.12a
3037	♂	07:02, 15:01	04:02	02:02	06:01	07:01:01	Hp-32.12b/73.12b
3065	♂	14:01	05:02, 04:04	10:04, 06:02:01	10:01, 06:02	06:01, 07:01:01	Hp-64.23/62.12a
3093	♀	08:01, 15:01	07:01:01, 04:02	02:02, 05:02	10:01, 06:01	06:01, 07:01:01	Hp-7.23/73.12b
3100	♂	08:05, 08:01	07:01:01, 06:01	05:02, 05:04	10:01, 06:01	06:01, 07:01:01	Hp-6.12b/7.23
3111	♀	15:01, 09:01^b^	07:01:02, 04:02	02:02, 05:03	10:01, 06:01	06:01, 07:01:01	Hp-28.23/73.12b
3112	♀	15:01, 09:01^b^	07:01:02, 04:02	02:02, 05:03	10:01, 06:01	06:01, 07:01:01	Hp-28.23/73.12b
3141	♀	14:01	04:04	06:02:01	06:02	07:01:01	Hp-62.12a
3216	♀	14:01, 12:01, 13:01	05:02, 04:04	10:01, 06:02:01	10:01, 06:02	06:01, 07:01:01	Hp-35.23/62.12a
3219	♀	14:01, 15:01, 09:01^b^	05:02, 07:01:02	10:04, 05:03	10:01, 08:01	06:01, 02:03	Hp-28.8b/64.23
3220	♀	15:01, 09:01^b^	07:01:02	05:03	10:01	06:01	Hp-28.23
3222	♀	15:01, 09:01^b^	07:01:02	05:03	10:01	06:01	Hp-28.23
3231	♂	08:01, 07:02	04:02, 07:01:01	02:02, 05:02	06:01, 08:01	07:01:01, 02:03	Hp-7.8b/32.12b
3241	♂	07:02, 15:01, 09:01^b^	04:02, 07:01:02	02:02, 05:03	06:01, 10:01	06:01, 07:01:01	Hp-28.23/32.12b
3242	♂	04:01:01, 07:02	04:01, 04:02	02:02, 04:02:01	05:01, 06:01	02:01, 07:01:01	Hp-4b.5/32.12b
3253	♀	08:01, 07:02	04:02, 07:01:01	02:02, 05:02	06:01, 08:01	07:01:01, 02:03	Hp-7.8b/32.12b
3255	♀	04:01:01, 08:01	07:01:01, 04:01	05:02, 04:02:01	05:01, 08:01	02:03, 02:01	Hp-4b.5/7.8b
3257	♀	08:01, 07:02	04:02, 07:01:01	02:02, 05:02	06:01, 08:01	07:01:01, 02:03	Hp-7.8b/32.12b
3313	♀	14:01	04:04	06:02:01	06:02	07:01:01	Hp-62.12a
**Yorkshire**							
4975	♀	08:08, 08:11	05:02	10:06, 10:05	10:01	06:01	Hp-26a.23/26b.23
5045	♀	04:01:01, 08:11	04:01, 05:02	10:06, 04:01	10:01, 02:01:01	06:01, 04:01:01	Hp-26b.23/4a.4a
5075	♂	04:01:01, 08:11	04:01, 05:02	10:06, 04:01	10:01, 02:01:01	06:01, 04:01:01	Hp-26b.23/4a.4a
5076	♂	04:01:01, 12:01, 13:01	04:01, 05:02	04:01, 10:01	02:01:01, 10:01	04:01:01, 06:01	Hp-4a.4a/35.23
5187	♀	01:01, 08:11	05:02, 01:01	01:01, 10:06	04:02, 10:01	02:02, 06:01	Hp-1a.15b/26b.23
5194	♂	04:01:01, 12:01, 13:01	04:01, 05:02	10:01, 04:01	10:01, 02:01:01	06:01, 04:01:01	Hp-4a.4a/35.23
5208	♀	11:03, 08:11, 08:12	05:02, 05:03:02	16:03, 10:06	09:01:01, 10:01	06:01, 09:01	Hp-26b.23/63.27
5227	♀	11:03, 08:11, 08:12	05:02, 05:03:02	16:03, 10:06	09:01:01, 10:01	06:01, 09:01	Hp-26b.23/63.27
5238	♀	04:01:01, 08:07	04:01, 03:06	04:01, 09:03	02:01:01	02:01, 04:01:01	Hp-4a.4a/58.2
5247	♀	08:08, 07:04	05:06, 05:02	10:05, 09:05	07:01, 10:01	02:01, 06:01	Hp-26a.23/37.24
5327	♂	04:01:01, 07:03	04:01, 06:01	05:05, 04:01	06:07, 02:01:01	02:12, 04:01:01	Hp-4a.4a/21.22
5341	♀	11:03, 08:11, 08:12	05:02, 05:03:02	16:03, 10:06	09:01:01, 10:01	06:01, 09:01	Hp-26b.23/63.27
5343	♀	11:03, 08:11, 08:12	05:02, 05:03:02	16:03, 10:06	09:01:01, 10:01	06:01, 09:01	Hp-26b.23/63.27
5344	♀	01:01, 08:11	01:01, 05:02	01:01, 10:06	04:02, 10:01	02:02, 06:01	Hp-1a.15b/26b.23
5346	♀	01:01, 08:11	01:01, 05:02	01:01, 10:06	04:02, 10:01	02:02, 06:01	Hp-1a.15b/26b.23

*^a^Designation refers to Table 3*.

^b^SLA-1*09:01 was detected by PCR-sequence specific primers but missed by mRNA SBT.

**Table 2:** **The alleles identified in Canadian specific pathogen-free Yorkshire and Landrace pigs which confirmed the unassigned alleles and standardized nomenclatures of the previously unconfirmed alleles in the GenBank database**.

Locus	Allele	GenBank accession number		Official International Society for Animal Genetics designation
		Submitted in this study	Previously submitted	
**The unassigned allele with complete coding sequence**				
*SLA-1*	SLA-1*dxbm05	KU754554	KX056221	SLA-1*07:04
	SLA-1*wzs01		KT194212	
	SLA-1*bm04		KT350995	
	–	KU754558	AK351685	SLA-1*08:11
*SLA-2*	–	KU754568	DQ992497	SLA-2*16:03
	–		DQ992498	
	–		DQ992499	
	–	KU754570	AK239592	SLA-2*10:06
**The unconfirmed allele with complete coding sequence**				
*SLA-1*	SLA-1*08pt13	KU953375	EU440337	SLA-1*08:07
	SLA-1*08sk11	KU754548	EU440332	SLA-1*08:08
	SLA-1*11jh02	KU754550	DQ883209	SLA-1*11:03
*SLA-2*	SLA-2*w09pt22	KU953376	EU432085	SLA-2*09:03
	SLA-2*10es21	KU754565	EU432090	SLA-2*10:04
	SLA-2*10sk21	KU754566	EU432083	SLA-2*10:05
*SLA-3*	SLA-3*03pt31	KU953377	EU432095	SLA-3*03:06
	SLA-3*hb06	KU754583	AP009559	SLA-3*04:04
**The unconfirmed allele with partial coding sequence**				
*SLA-1*	SLA-1*rh03	KU754555	AF074427	SLA-1*07:03
*SLA-2*	SLA-2*05rh03	KU754569	AF074428	SLA-2*05:05
*DRB1*	DRB1*06sL47	KU754601	L08847	DRB1*06:07
*DQB1*	DQB1*0203	KU754591	AB012093	DQB1*02:03
			AF113970	
	DQB1*0204	KU754590	L08844	DQB1*02:12
			EU039916	

**Figure 1:**
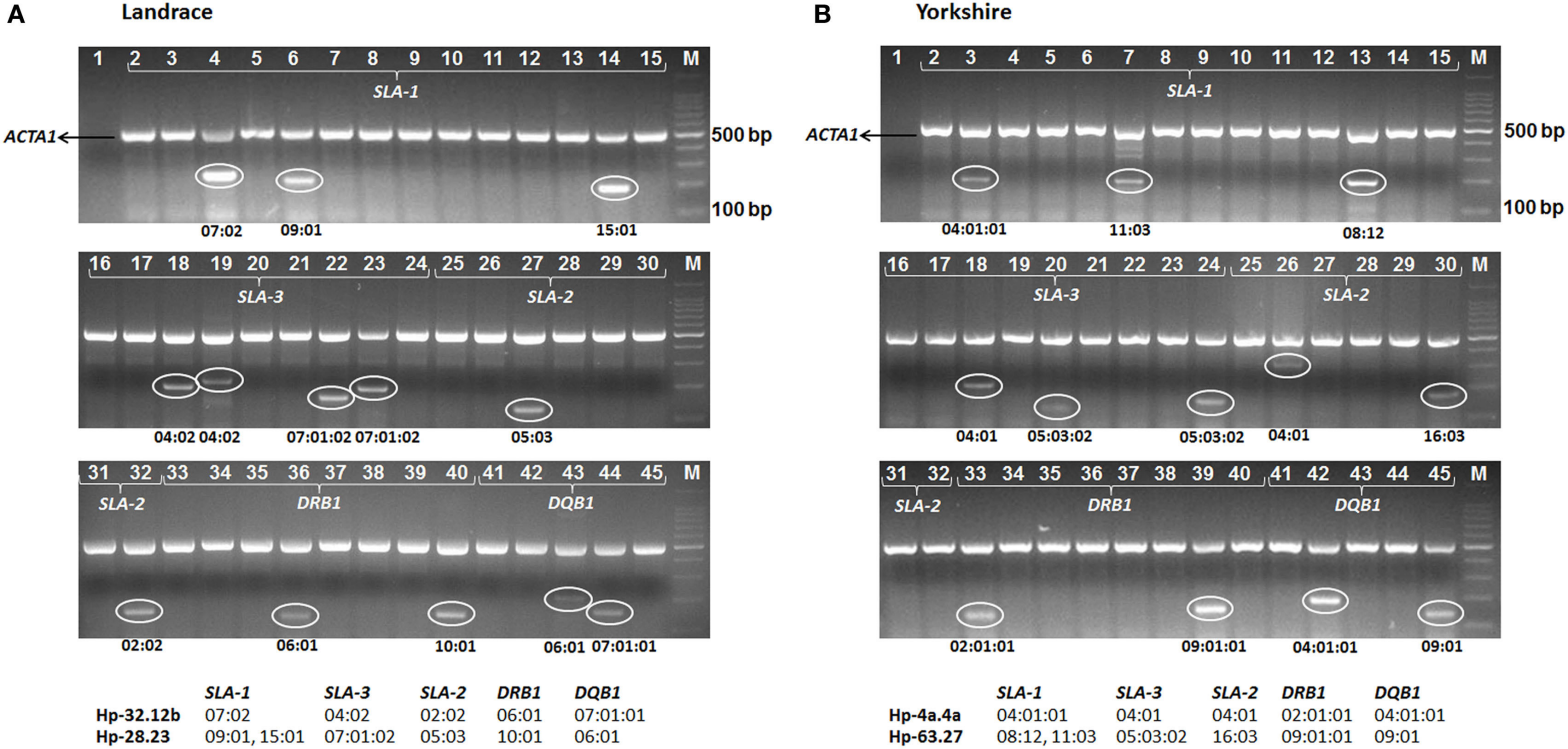
**Examples of swine leukocyte antigen (SLA) genotyping in Canadian specific pathogen-free Yorkshire and Landrace pigs at five loci (*SLA-1, SLA-3, SLA-2, DRB1*, and *DQB1*) by PCR-sequence-specific primers using the full primer set (44 primer pairs, lane 2 through 45, specific for each alleles identified in pigs) (Table S2 in Supplementary Material)**. Negative controls with all reagents except DNA were used to check for contamination (lane 1). Positive internal control primers (porcine *ACTA1*, 516 bp) were multiplexed into each reaction to check for adequate amplification (upper band). The presence of a smaller PCR product indicates the sample was positive for the allele(s) in the corresponding reaction (lower band). Product sizes of each allele-specific primer are given in Table S2 in Supplementary Material. Pig samples shown here are [**(A)** Landrace] SLA Hp-32.12b/28.23 (Table 3), with specific amplifications in lanes 4 (SLA-1*07:02), 6 (SLA-1*09:01), 14 (SLA-1*15:01), 18 (SLA-3*04:02), 19 (SLA-3*04:02), 22 (SLA-3*07:01:02), 23 (SLA-3*07:01:02), 27 (SLA-2*05:03), 32 (SLA-2*02:02), 36 (DRB1*06:01), 40 (DRB1*10:01), 43 (DQB1*06:01), and 44 (DQB1*07:01:01); and [**(B)** Yorkshire] SLA Hp-4a.4a/63.27 (Table 3), with specific amplifications in lanes 3 (SLA-1*04:01:01), 7 (SLA-1*11:03), 13 (SLA-1*08:12), 18 (SLA-3*04:01), 20 (SLA-3*05:03:02), 24 (SLA-3*05:03:02), 26 (SLA-2*04:01), 30 (SLA-2*16:03), 33 (DRB1*02:01:01), 39 (DRB1*09:01:01), 42 (DQB1*04:01:01), and 45 (DQB1*09:01). *M*, 100 bp PCR marker (TaKaRa, Dalian, China).

Based on the SLA alleles identified in the parental SPF Yorkshire and Landrace pigs, 44 pairs of sequence-specific typing primers were designed to differentiate each of the 62 alleles (including SLA-1*09:01 allele) using PCR (Table S2 in Supplementary Material). The results of PCR-SSP SLA typing of the 37 founding pigs were consistent with the SBT data (Table 1). Subsequently, all F1 progeny pigs were genotyped using the complete set of primers to assign alleles to haplotypes based on inheritance and segregation patterns by descent. A representative gel image showing the typing of Yorkshire and Landrace pigs is shown in Figure 1. The SLA genotypes of each individual were determined according to the allele-specific bands. The results showed that a total of 19 SLA haplotypes were identified in SPF Yorkshire and Landrace pigs at the molecular level, including 17 class I haplotypes and 11 class II haplotypes. Official ISAG haplotype designations were assigned by the SLA Nomenclature Committee (Table 3). Among these, three class I haplotypes, Hp-37.0, 63.0, and 73.0, appeared to be novel and have not previously been reported in other pig populations (Table 4). The most common haplotype Hp-26b.23 (SLA-1*08:11–SLA-3*05:02–SLA-2*10:06–DRB1*10:01–DQB1*06:01) and Hp-32.12b (SLA-1*07:02–SLA-3*04:02–SLA-2*02:02–DRB1*06:01–DQB1*07:01:01) was identified in 76 Yorkshire and 74 Landrace pigs with frequencies of 29.7 and 20.7%, respectively. Two *SLA-1* alleles were observed in three class I haplotypes in pigs: Hp-28.0 (SLA-1*09:01, SLA-1*15:01), Hp-35.0 (SLA-1*12:01, SLA-1*13:01), and Hp-63.0 (SLA-1*08:12, SLA-1*11:03). Several haplotypes appeared to be the result of crossovers between class I and class II regions, e.g., Hp-7.8b, comprising class I haplotype Hp-7.23 and class II haplotype Hp-28.8b. Hp-28.8b and 28.23 haplotypes comprised different sets of class II alleles but shared the same class I alleles. Hp-7.23, 26a.23, 26b.23, 28.23, 35.23, and 64.23 haplotypes comprised different sets of class I alleles but shared the same class II alleles.

**Table 3:** **Swine leukocyte antigen (SLA) haplotypes and corresponding alleles identified in Canadian specific pathogen-free Yorkshire and Landrace pigs**.

SLA haplotype [International Society for Animal Genetics (ISAG) Nomenclature]	Class I loci			Class II loci		No. of pigs/haplotype frequency (%)			
	*SLA-1*	*SLA-3*	*SLA-2*	*DRB1*	*DQB1*	Y^a^	L^a^	Y × L^a^	L × Y^a^

						2*n* = 256	2*n* = 358	2*n* = 62	2*n* = 58
Hp-1a.15b	01:01	01:01	01:01	04:02	02:02	25/9.8			
Hp-4a.4a	04:01:01	04:01	04:01	02:01:01	04:01:01	67/26.2		17/27.4	9/15.5
Hp-4b.5	04:01:01	04:01	04:02:01	05:01	02:01		26/7.3		
Hp-6.12b	08:05	06:01	05:04	06:01	07:01:01		26/7.3		
Hp-7.8b	08:01	07:01:01	05:02	08:01	02:03		36/10.0	5/8.1	
Hp-7.23	08:01	07:01:01	05:02	10:01	06:01		31/8.6		
Hp-21.22	07:03	06:01	05:05	06:07	02:12	18/7.0		4/6.5	
Hp-26a.23	08:08	05:02	10:05	10:01	06:01	7/2.7			7/12.1
Hp-26b.23	08:11	05:02	10:06	10:01	06:01	76/29.7	12/3.4		13/22.4
Hp-28.8b	09:01^b^, 15:01	07:01:02	05:03	08:01	02:03		6/1.7		
Hp-28.23	09:01^b^, 15:01	07:01:02	05:03	10:01	06:01		48/13.4	11/17.7	14/24.1
Hp-32.12b	07:02	04:02	02:02	06:01	07:01:01		74/20.7	4/6.5	15/25.9
Hp-35.23	12:01, 13:01	05:02	10:01	10:01	06:01	19/7.4	6/1.7	10/16.1	
Hp-37.24	07:04	05:06	09:05	07:01	02:01	11/4.3			
Hp-58.2	08:07	03:06	09:03	02:01:01	02:01	6/2.3			
Hp-62.12a	14:01	04:04	06:02:01	06:02	07:01:01		27/7.5	10/16.1	
Hp-63.27	08:12, 11:03	05:03:02	16:03	09:01:01	09:01	26/10.2			
Hp-64.23	14:01	05:02	10:04	10:01	06:01		33/9.2	1/1.6	
Hp-73.12b	15:01	04:02	02:02	06:01	07:01:01		31/8.6		
**Recombinant haplotype**									
Hp-*NA*.12b^c^	09:01^b^, 15:01	04:02	02:02	06:01	07:01:01		1/0.3		
Hp-64.8b	14:01	05:02	10:04	08:01	02:03		1/0.3		
Hp-26b.4a	08:11	05:02	10:06	02:01:01	04:01:01	1/0.4			

*^a^Y, Yorkshire; L, Landrace; Y × L, Yorkshire Landrace cross; L × Y, Landrace Yorkshire cross*.

^b^SLA-1*09:01 was detected by PCR-sequence-specific primers but missed by mRNA sequence-based typing.

*^c^NA, official designation of the crossover class I haplotypes was not assigned by the ISAG SLA Nomenclature Committee*.

**Table 4:** **Swine leukocyte antigen (SLA) class I and class II haplotypes identified in Canadian specific pathogen-free Yorkshire and Landrace pigs and in swine breeds and cell lines with published identical haplotypes**.

SLA class I haplotype	Breed^a^	Reference	SLA class II haplotype	Breed^a^	Reference
Hp-1a.0	Large white	Renard et al. (25)	Hp-0.2	Landrace × Yorkshire	Le et al. (26)
	KSU, PCV, Big pig^b^	Ho et al. (16)		Sinclair, Hanford	Ho et al. (27)
	Pietrain^b^	Essler et al. (31)		NIH	Smith et al. (28)
	Danish pigs^b^	Pedersen et al. (19)		MPK	Ho et al. (21)
	German Landrace^b^	Gimsa et al. (32)		PCV, Big pig, KSU^b^	Ho et al. (17)
				SNU	Yeom et al. (29)
				Du, Y × L, La, NIH, Os, SNU, Yo	Le et al. (30)

Hp-4a.0	NIH Duroc Meishan^c^	Oleksiewicz et al.; Smith et al. (5, 28) Immuno Polymorphism Database (IPD)-MHC database Ho et al. (14)	Hp-0.4a	Landrace × Yorkshire NIH	Le et al. (26) Smith et al. (28)
Hp-4b.0	PK13, PK15	Ho et al. (21)		Big pig^b^	Ho et al. (17)
	Yucatan	Smith et al. (28)		Y × L, NIH	Le et al. (30)
Hp-4.0	KSU, PCV, Big pig^b^ Danish pigs^b^ Yucatan^b^ German Landrace^b^	Ho et al. (16) Pedersen et al. (19) Choi et al. (33) Gimsa et al. (32)			

Hp-6.0	Microminipig	Ando et al. (34)	Hp-0.5	Landrace × Yorkshire	Le et al. (26)
	Yucatan	Smith et al.; Choi et al. (28, 33)		PK13, PK15	Ho et al. (21)
	Big pig^b^	Ho et al. (16)		Yucatan	Smith et al.; Choi et al. (28, 33)
	Danish pigs^b^	Pedersen et al. (19)		PCV^b^	Ho et al. (17)
	German Landrace^b^	Gimsa et al. (32)		Y × L, La, Os, Yo	Le et al. (30)

Hp-7.0	Korean native pig^b^	Cho et al. (35)	Hp-0.8b	Landrace × Yorkshire	Le et al. (26)
	Yucatan	Smith et al. (28)		PCV^b^	Ho et al. (17)
	PCV^b^	Ho et al. (16)		Y × L	Le et al. (30)
	Danish pigs^b^	Pedersen et al. (19)		German Landrace^b^	Gimsa et al. (32)
	German Landrace^b^	Gimsa et al. (32)			

Hp-21.0	Commercial breeds	IPD-MHC database	Hp-0.12a	Sinclair	Ho et al. (27)
	PCV, Big pig^b^	Ho et al. (16)	Hp-0.12	Pietrain^b^	Essler et al. (31)
				PCV, Big pig^b^	Ho et al. (17)
				German Landrace^b^	Gimsa et al. (32)

Hp-26a.0	SK-RST	Ho et al. (21)	Hp-0.15b	Landrace × Yorkshire	Le et al. (26)
Hp-26.0	KSU^b^	Ho et al. (16)		Banna	Zeng et al.; Zeng and Zeng (36, 37)
	Pietrain^b^	Essler et al. (31)		PCV, Big pig, KSU^b^	Ho et al. (17)
	Danish pigs^b^	Pedersen et al. (19)		Ld, Yo	Le et al. (30)
	German Landrace^b^	Gimsa et al. (32)		

Hp-28.0	Landrace	Tanaka-Matsuda et al. (20)	Hp-0.22	PCV, Big pig, MY^b^	Ho et al. (17)
	Pietrain^b^	Essler et al. (31)			
	German Landrace^b^	Gimsa et al. (32)			

Hp-32.0	IU-pig model	Reyes et al. (38)	Hp-0.23	Microminipig	Ando et al. (34)
	ST	Ho et al. (21)		IU-pig model	Reyes et al. (38)
	Big pig^b^	Ho et al. (16)		Landrace × Yorkshire	Le et al. (26)
	Pietrain^b^	Essler et al. (31)		Guizhou	Liu et al. (39)
	Danish pigs^b^	Pedersen et al. (19)		Korean native pig^b^	Cho et al. (35)
	German Landrace^b^	Gimsa et al. (32)		Big pig^b^	Ho et al. (17)
				Pietrain^b^	Essler et al. (31)
				Y × L, Yo	Le et al. (30)
				German Landrace^b^	Gimsa et al. (32)

Hp-35.0	Bama	Gao et al. (15)	Hp-0.24	Landrace × Yorkshire	Le et al. (26)
	Microminipig	Ando et al. (34)		Big pig^b^	Ho et al. (17)
	IU-pig model	Reyes et al. (38)		Pietrain^b^	Essler et al. (31)
	Pietrain^b^	Essler et al. (31)		AGH, Y × L, Yo	Le et al. (30)
	PT-K75	Ho et al. (21)			
	German Landrace^b^	Gimsa et al. (32)			

Hp-58.0	PT-K75	Ho et al. (21)	Hp-0.27	Landrace × Yorkshire	Le et al. (26)
				Guizhou	Liu et al. (39)
				PCV^b^	Ho et al. (17)
				Du, Ld, Y × L	Le et al. (30)

Hp-62.0	Landrace	Tanaka-Matsuda et al. (20)			
	Danish pigs^b^	Pedersen et al. (19)			
	German Landrace^b^	Gimsa et al. (32)			

Hp-64.0	ESK-4	Ho et al. (21)			
	Pietrain^b^	Essler et al. (31)			

*^a^Breed in which the haplotype has been reported*.

*^b^Low-resolution SLA haplotype*.

*^c^Hp-4c.0 differs from Hp-4a.0 at the SLA-6 locus*.

**Figure 2:**
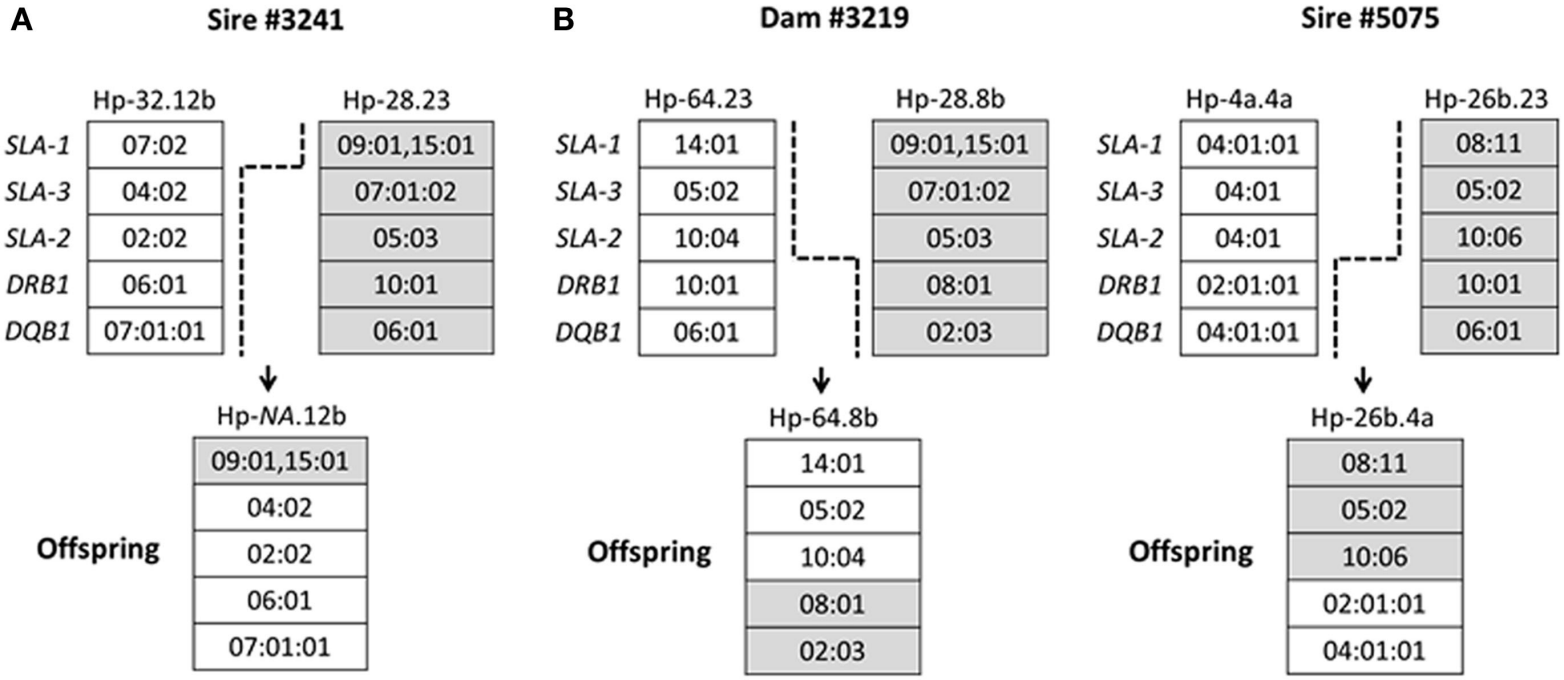
**Schematic diagram of swine leukocyte antigen (SLA) crossovers identified in Canadian specific pathogen-free Yorkshire and Landrace pigs**. Five loci (*SLA-1, SLA-3, SLA-2, DRB1*, and *DQB1*) of the SLA complex are shown. *Broken lines* indicate probable positions where the crossover occurred. Panel **(A)** indicates one crossover within the class I region; panel **(B)** indicates two crossovers between class I and class II regions. *NA*, official designation of crossover class I haplotypes not assigned by the International Society for Animal Genetics SLA Nomenclature Committee.

Among the 367 pigs SLA genotyped in this study, a total of three crossovers were identified within the SLA region. The details of each crossover are shown schematically in Figure 2. One crossover occurred within the class I region, resulting in one new recombinant haplotype (Figure 2A), with a haplotype frequency of 0.3%. Two crossovers involved exchanges between class I and class II regions, resulting in two new recombinant haplotypes Hp-64.8b and Hp-26b.4a (Figure 2B), with haplotype frequencies of 0.3 and 0.4%, respectively.

### SLA Class I Molecule Clustering

**Figure 3:**
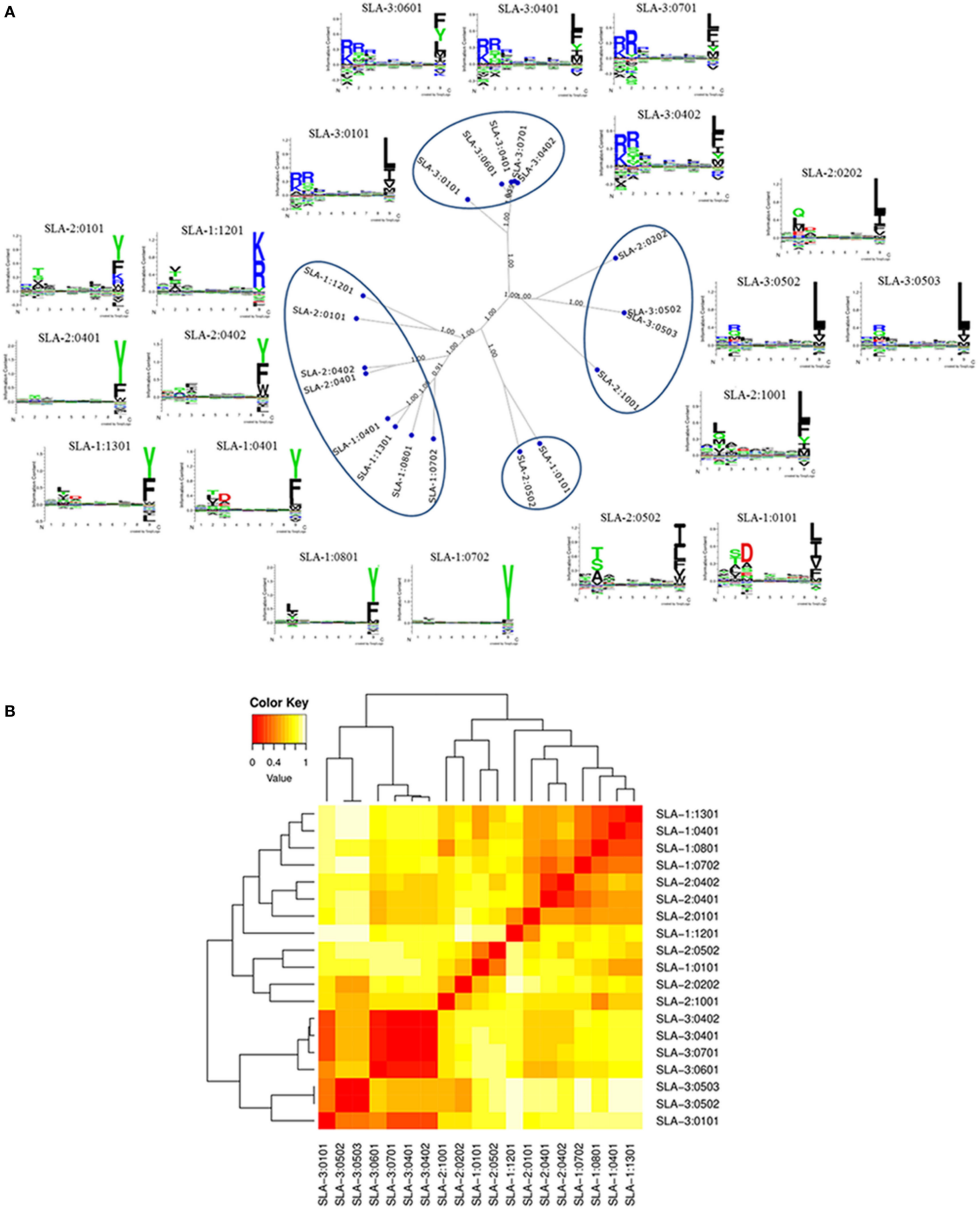
**Functional clustering of 19 identified frequent swine leukocyte antigen (SLA) class I alleles in Canadian specific pathogen-free Yorkshire and Landrace pigs using *MHCcluster***. Panel **(A)** shows a tree representation of the clustering; panel **(B)** shows a heat map representation. The tree was visualized using the advanced tree-viewer in *MHCcluster*. Sequence logos of the predicted binding specificity are included for each class I allele.

To illustrate the important differences between sequence- and function-based clustering, we generated clusters of SLA class I alleles by functional clustering using the *MHCcluster* method. Nineteen identified frequent class I alleles in Yorkshire and Landrace pigs were chosen for analysis, including six *SLA-1* alleles, six *SLA-2* alleles, and seven *SLA-3* alleles, which already exists in the MHC allele lists of *MHCcluster* Server. *MHCcluster* analysis was based on the comparison of predicted binding motifs for each individual SLA molecule and gave an unrooted tree visualization of functional peptide-binding similarities. This analysis demonstrated that clustering to a very high degree produced four distinct specificity groups (Figure 3). However, some SLA class I molecules were not well characterized by the specificities of four supertypes. Each of four clusters consisted of SLA class I molecules with highly divergent specificities, and some molecules, e.g., SLA-1*12:01, SLA-3*01:01, SLA-2*02:02, and SLA-2*10:01, were poorly characterized by the specificities of four supertypes. Moreover, the sequence homologies between SLA-1*01:01 and SLA-2*05:02 molecules were 89.7% at the amino acid level, but examination of the binding specificities represented by the sequence logos that described the predicted binding motif for each SLA molecule revealed that SLA-1*01:01 and SLA-2*05:02 molecules were functionally similar compared with other molecules, e.g., SLA-1*04:01 and SLA-1*13:01, which shared 97.3% similarity at the amino acid level. The peptide-binding motifs of SLA-3*01:01 was shown to have a mixed SLA-3*04:02 and SLA-3*05:02/05:03 specificity matching SLA-3*04:02 at the N-terminal and SLA-3*05:02/05:03 at the C-terminal ends. These molecules all had predicted accuracy values of 1.00, indicating that the molecules were well characterized by the peptide-binding data. The calculated binding motifs were thus considered to have a high likelihood of giving a correct representation of the specificity of the molecules. In addition, although SLA-1*04:01 and SLA-1*13:01 molecules shared close to 97.3% similarity, differing by only nine amino acid substitutions, they appeared to have functional differences, despite being classified in a single cluster using functional clustering.

## Discussion

Specific pathogen-free Yorkshire and Landrace pigs were imported from Canadian Genesus incorporated in 2014 and represent the most important large animal model for studying host immune responses to viruses in China. The SLA complex consists of many important immune-related genes and has been repeatedly associated with variations in immunological and physiological performances. Therefore, it is essential to define the SLA alleles and haplotypes of these pigs molecularly, to use them preferably as classical laboratory animals for clarifying the molecular mechanisms responsible for host immune responses. In this study, we identified and assigned 43 class I and 18 class II alleles at five SLA loci representing 17 class I haplotypes and 11 class II haplotypes, by the SBT and PCR-SSP methods. The official ISAG haplotype designations were assigned by the SLA Nomenclature Committee. These submissions confirmed and defined many previously unconfirmed alleles and haplotypes. The SLA Nomenclature Committee adopted the naming protocol for human leukocyte antigen alleles and has converted the SLA nomenclature system to better resemble the human leukocyte antigen system. Currently, the confirmed alleles in this study have been released in updated IPD-MHC SLA database [Release 2.0.0.3 (2016-11-03)]. Among these haplotypes, three class I haplotypes, Hp-37.0, 63.0, and 73.0, appeared to be novel and have not previously been reported in other pig breeds, although the SLA-1*07:04 allele was found in the Chinese Bama miniature pig (KX056221) and Wuzhishan miniature pig (KT194212), and the SLA-2*16:03 allele was obtained from the clone KNP2006 (DQ992497)/KNP2007 (DQ992498)/KNP2008 (DQ992499), constructed from Korean native pigs. This suggests that these pigs might also carry the corresponding haplotypes. Inheritance and segregation patterns by descent demonstrated the presence of a duplicated *SLA-1* locus in Hp-28.0, Hp-35.0, and Hp-63.0. In particular, the SLA-1*09:01 allele was not detected by SBT method. However, it has been found that the SLA-1a*09:01 and SLA-1b*15:01 alleles of Hp-28.0 are mapped between a 149-kb genomic segment of the SLA class I gene clusters by BAC sequencing analyses (20), and our results of PCR-SSP SLA typing were consistent with the previous findings.

Haplotype Hp-4.0 (4a.0 or 4b.0) was the more prevalent class I haplotype in Yorkshire, Landrace, and crossbred progeny between Yorkshire and Landrace pigs. This is consistent with previous reports identifying Hp-4.0 as commonly present in swine breeds worldwide, including NIH, Duroc, Meishan, Yucatan, Danish pigs, German purebred Landrace, and outbred pig populations, and “PK13, PK15” cell lines (5, 14, 16, 19, 21, 28, 32, 33), indicating that Hp-4.0 is a valuable SLA class I haplotype that has survived long-term evolutionary selection. No novel class II haplotypes were identified in SPF Yorkshire and Landrace pigs. Hp-0.23 was the most common SLA class II haplotype in this study and was shared by several other pig populations, including Microminipigs, the IU-pig model, Landrace × Yorkshire, Guizhou miniature pigs, Korean native pigs, Big pigs, German purebred Landrace, and Pietrain pigs (17, 26, 31, 32, 34, 35, 38, 39) (Table 4). This suggests that Yorkshire and Landrace pigs may share some common genetic background with these breeds.

Six SLA class I haplotypes, Hp-4b.0, Hp-26a.0, Hp-32.0, Hp-35.0, Hp-58.0, and Hp-64.0, identified in this study had been also identified in six commercially available porcine cell lines, ESK-4, PK13, PK15, PT-K75, SK-RST, and ST (21). In addition, two class I haplotypes (Hp-32.0 and Hp-35.0) and a class II haplotype (Hp-0.23) had also been identified in the crossbred IU-pig model for use in xenotransplant studies (38). This suggests that Yorkshire or Landrace pigs might have been commonly used to involve in the establishment of some porcine cell lines and specific animal models.

We also found recombination events in the SLA region. One recombinant within the SLA class I region and two between class I and class II regions were identified in 367 Yorkshire and Landrace pigs. Three SLA recombinants were previously identified in 281 Chinese Bama miniature pigs (15), and six crossovers were also identified within the SLA region in 466 Sinclair and Hanford pigs (27). A novel line of miniature pigs, Microminipigs, which have a considerably smaller body size than other miniature pig breeds, such as Clawn, Yucatan, NIH, Sinclair, and Hanford, also have three recombinant haplotypes (34). This suggests the existence of recombination hotspots in the SLA class I and class III regions. Crossover within the SLA class II region has not yet been found. Importantly, these pig breeds will be useful for studying the effects of selected differences at SLA class I and/or class II loci on parameters of transplantation immunity.

Yorkshire and Landrace pig breeds are distributed worldwide, and this study may not have fully revealed the extent of SLA diversity in these breeds because of specific selection in different countries, the possibility of founder effects, the relatively small sample size, and the breeding schemes for Yorkshire and Landrace pigs. However, the results provide the first data for the SLA haplotype repertoire of SPF Yorkshire and Landrace pigs and improve our knowledge of the allelic architecture of the SLA system. Previous studies have also shown that the SLA protein can inhibit natural killer-mediated cytotoxicity (40). Therefore, several resource populations of pigs with well-characterized SLA antigens are now available worldwide for experiments in various disciplines, with special emphasis on xenotransplantation and immunologic mechanism studies.

The peptide-binding function of only seven already known SLA class I alleles identified in Danish swine herds have been analyzed using the *MHCcluster* method (19). The functional diversity of the majority of SLA class I alleles have never been comprehensively compared up to now. Canadian SPF Yorkshire and Landrace pigs are more suitable as laboratory animal models for biomedical research because of their unique advantages. In order to obtain more extensive applications for these two swine species and decide which SLA proteins to include in future functional analyses, it is significant and necessary to comparative analyze the functional diversity of SLA class I alleles identified in this study. We chose 19 identified frequent SLA class I alleles in SPF Yorkshire and Landrace pigs for clustering analysis using the *MHCcluster* method. Even though the application of this method is limited to comparing functional similarities between large sets of MHC molecules, it is an effective visual tool combining functional clustering and visualization of predicted binding motifs, allowing for direct functional mapping of MHC molecules. Most of the polymorphism of SLA class I proteins is resident in the peptide-binding groove. Crystal structural analysis indicated that the peptide-binding groove of SLA class I molecules contains six pockets and these pockets play critical biochemical roles in determining the peptide-binding motif of SLA class I alleles (6, 41). SLA molecules perform the important functions of sampling and displaying peptides to T cells. The binding of an appropriate peptide to SLA is the single most selective event in antigen presentation. Any search for immunogenic epitopes should therefore consider the identification of SLA-binding peptides to be a high priority. However, the binding of different SLA alleles and different classes of peptide was determined by the fit between these “pockets” and “anchor” residues in the peptides (4–6). Only a fraction of peptides that fit the simple kind of motif used actually bind the corresponding class I molecule with measurable affinities. Therefore, the characterization of the peptide-binding motifs of SLA class I proteins is beneficial to identify the cognate virally derived peptides and made it possible to explore the nature of the SLA proteins and the roles they play in establishing adaptive immunity. It could also lead to accelerated development of peptide vaccines with increased efficacy due to optimal activation of cell-mediated immune responses with minimal adverse events. Moreover, the peptide motifs of MHC molecules can determine the resistance and susceptibility to some important animal pathogen, for example, for the dominantly expressed chicken MHC class I molecule (42). In this study, we confirmed the existence of four supertypes to analyze the specificities of 19 SLA class I molecules. However, the analysis also clearly revealed that not all class I molecules fitted equally well into a supertype classification scheme, and that each supertype consisted of class I molecules with highly divergent specificities. It demonstrate that common SLA class I proteins have high levels of functional diversity. The binding specificities represented by the sequence logos indicated that some different alleles (e.g., SLA-3*01:01, SLA-3*04:01, SLA-3*04:02, and SLA-3*07:01) appeared to be similar in terms of function and were classified in a single cluster using functional clustering. This indicated a somewhat shared functionality among the SLA class I proteins SLA-3*01:01, SLA-3*04:01, SLA-3*04:02, and SLA-3*07:01 compared with other proteins (e.g., SLA-1*01:01 and SLA-2*05:02), and distinct from the others. It suggests that clustering analysis could be as a guide to help us understanding immunological phenotypic similarities between pig breeds using information about SLA types, especially for some pigs heterozygous at SLA class I loci. It could also provide information on where a specific allele (maybe present at a high frequency in a particular cohort) fits into the specificity space covered by the common MHC molecules. Furthermore, clustering analysis revealed functional differences among some SLA class I proteins, which suggests that we can produce these molecules to broaden the repertoire and achieve a higher chance of matching animals in terms of SLA expression in future peptide–epitope studies. The results of the current study thus demonstrate that the MHC clustering method can be used to identify specific MHC proteins with the most diverse and broad coverage of the overall peptide-binding repertoire and chance for cytotoxic lymphocyte recognition.

In conclusion, we molecularly defined 17 class I haplotypes and 11 class II haplotypes in Canadian SPF Yorkshire and Landrace pigs using SBT and PCR-SSP methods. The official ISAG haplotype designations were assigned by the SLA Nomenclature Committee. These pig breeds with known SLA backgrounds will be valuable experimental animals for animal health and biomedical research, including swine immune responses and transplantation studies. We also have analyzed the functional diversities of 19 identified frequent SLA class I molecules and confirmed the existence of four supertypes using the *MHCcluster* method. This information is important for investigating immunological phenotypic differences, screening potential T-cell epitopes, and studying T-cell immune responses to specific epitopes of certain endemic pathogens.

## Author Contributions

CG and HC designed the experiments, analyzed the data, and wrote the paper. CG, JQ, and XJ performed the experiments. CL and XL collected samples.

## Conflict of Interest Statement

The authors declare that the research was conducted in the absence of any commercial or financial relationships that could be construed as a potential conflict of interest.

## Footnotes

^1^http://www.ebi.ac.uk/ipd/mhc/group/SLA, Release 2.0.0.3 (2016-11-03).

^2^http://sb.nhri.org.tw/ICES.

^3^http://www.cbs.dtu.dk/services/MHCcluster/.

## Supplementary Material

Table S1 **The pedigree of Canadian specific pathogen-free (SPF) Yorkshire and Landrace pigs with their respective check numbers assigned by Harbin Veterinary Research Institute**.

Table S2 **Specificities of sequence-specific PCR primers for rapid swine leukocyte antigen (SLA) typing of Canadian specific pathogen-free (SPF) Yorkshire and Landrace pigs**.

Data Sheet 1 **The alignments of the nucleotide sequences including the complete coding sequences of 61 alleles identified in Canadian specific pathogen-free Yorkshire and Landrace pigs at five swine leukocyte antigen (SLA) loci**.

## Abbreviations

MHC, major histocompatibility complex; SLA, swine leukocyte antigen; SPF, specific pathogen-free; RT-PCR, the reverse transcription polymerase chain reaction; SBT, sequence-based typing; PCR-SSP, PCR-sequence specific primers; IPD, the Immuno Polymorphism Database; ISAG, International Society for Animal Genetics.

## References

[1] Renard C Hart E Sehra H Beasley H Coggill P Howe K The genomic sequence and analysis of the swine major histocompatibility complex. Genomics (2006) 88:96–110. 10.1016/j.ygeno.2006.01.004 16515853

[2] Neefjes J Jongsma ML Paul P Bakke O. Towards a systems understanding of MHC class I and MHC class II antigen presentation. Nat Rev Immunol (2011) 11:823–36. 10.1038/nri3084 22076556

[3] Lunney JK Ho CS Wysocki M Smith DM. Molecular genetics of the swine major histocompatibility complex, the SLA complex. Dev Comp Immunol (2009) 33:362–74. 10.1016/j.dci.2008.07.002 18760302

[4] Gao F Fang Q Li Y Li X Hao H Xia C. Reconstruction of a swine SLA-I protein complex and determination of binding nonameric peptides derived from the foot-and-mouth disease virus. Vet Immunol Immunopathol (2006) 113:328–38. 10.1016/j.vetimm.2006.06.002 16870265

[5] Oleksiewicz M Kristensen B Ladekjaer-Mikkelsen AS Nielsen J. Development of a rapid in vitro protein refolding assay which discriminates between peptide-bound and peptide-free forms of recombinant porcine major histocompatibility class I complex (SLA-I). Vet Immunol Immunopathol (2002) 86:55–77. 10.1016/S0165-2427(02)00015-6 11943330

[6] Zhang N Qi J Feng S Gao F Liu J Pan X Crystal structure of swine major histocompatibility complex class I SLA-1* 0401 and identification of 2009 pandemic swine-origin influenza A H1N1 virus cytotoxic T lymphocyte epitope peptides. J Virol (2011) 85:11709–24. 10.1128/JVI.05040-11 21900158 PMC3209268

[7] Wang Y Zhou Y Li G Zhang S Jiang Y Xu A Identification of immunodominant T-cell epitopes in membrane protein of highly pathogenic porcine reproductive and respiratory syndrome virus. Virus Res (2011) 158:108–15. 10.1016/j.virusres.2011.03.018 21458510

[8] Pedersen LE Harndahl M Rasmussen M Lamberth K Golde WT Lund O Porcine major histocompatibility complex (MHC) class I molecules and analysis of their peptide-binding specificities. Immunogenetics (2011) 63:821–34. 10.1007/s00251-011-0555-3 21739336 PMC3214623

[9] Liao Y Lin H Lin C Chung W. Identification of cytotoxic T lymphocyte epitopes on swine viruses: multi-epitope design for universal T cell vaccine. PLoS One (2013) 8:e84443. 10.1371/journal.pone.0084443 24358361 PMC3866179

[10] Gerner W Kaser T Saalmuller A Porcine T lymphocytes and NK cells – an update. Dev Comp Immunol (2009) 33:310–20. 10.1016/j.dci.2008.06.003 18601948

[11] Pedersen LE Rasmussen M Harndahl M Nielsen M Buus S Jungersen G A combined prediction strategy increases identification of peptides bound with high affinity and stability to porcine MHC class I molecules SLA-1*04:01, SLA-2*04:01, and SLA-3*04:01. Immunogenetics (2016) 68:157–65. 10.1007/s00251-015-0883-9 26572135

[12] Gutierrez AH Loving C Moise L Terry FE Brockmeier SL Hughes HR In vivo validation of predicted and conserved T cell epitopes in a swine influenza model. PLoS One (2016) 11:e0159237. 10.1371/journal.pone.0159237 27411061 PMC4943726

[13] Thomsen M Lundegaard C Buus S Lund O Nielsen M. MHCcluster, a method for functional clustering of MHC molecules. Immunogenetics (2013) 65:655–65. 10.1007/s00251-013-0714-9 23775223 PMC3750724

[14] Ho CS Rochelle ES Martens GW Schook LB Smith DM. Characterization of swine leukocyte antigen polymorphism by sequence-based and PCR-SSP methods in Meishan pigs. Immunogenetics (2006) 58:873–82. 10.1007/s00251-006-0145-y 17039361

[15] Gao C Jiang Q Guo D Liu J Han L Qu L. Characterization of swine leukocyte antigen (SLA) polymorphism by sequence-based and PCR-SSP methods in Chinese Bama miniature pigs. Dev Comp Immunol (2014) 45:87–96. 10.1016/j.dci.2014.02.006 24560654

[16] Ho CS Lunney J Franzo-Romain M Martens G Lee YJ Lee JH Molecular characterization of swine leucocyte antigen class I genes in outbred pig populations. Anim Genet (2009) 40:468–78. 10.1111/j.1365-2052.2009.01860.x 19392823

[17] Ho CS Lunney J Lee JH Franzo-Romain M Martens G Rowland R Molecular characterization of swine leucocyte antigen class II genes in outbred pig populations. Anim Genet (2010) 41:428–32. 10.1111/j.1365-2052.2010.02019.x 20121817

[18] Lee Y Cho K Kim M Smith D Ho C Jung K Sequence-based characterization of the eight SLA loci in Korean native pigs. Int J Immunogenet (2008) 35:333–4. 10.1111/j.1744-313X.2008.00775.x 18549392

[19] Pedersen LE Jungersen G Sorensen MR Ho C-S Vadekaer DF. Swine leukocyte antigen (SLA) class I allele typing of Danish swine herds and identification of commonly occurring haplotypes using sequence specific low and high resolution primers. Vet Immunol Immunopathol (2014) 162:108–16. 10.1016/j.vetimm.2014.10.007 25457547

[20] Tanaka-Matsuda M Ando A Rogel-Gaillard C Chardon P Uenishi H. Difference in number of loci of swine leukocyte antigen classical class I genes among haplotypes. Genomics (2009) 93:261–73. 10.1016/j.ygeno.2008.10.004 18996466

[21] Ho C Franzo-Romain M Lee Y Lee J Smith D. Sequence-based characterization of swine leucocyte antigen alleles in commercially available porcine cell lines. Int J Immunogenet (2009) 36:231–4. 10.1111/j.1744-313X.2009.00853.x 19508353

[22] Smith D Lunney J Ho CS Martens G Ando A Lee JH Nomenclature for factors of the swine leukocyte antigen class II system, 2005. Tissue Antigens (2005) 66:623–39. 10.1111/j.1399-0039.2005.00492.x 16305679

[23] Smith D Lunney J Martens G Ando A Lee JH Ho CS Nomenclature for factors of the SLA class-I system, 2004. Tissue Antigens (2005) 65:136–49. 10.1111/j.1399-0039.2005.00337.x 15713212

[24] Ho CS Lunney J Ando A Rogel-Gaillard C Lee JH Schook L Nomenclature for factors of the SLA system, update 2008. Tissue Antigens (2009) 73:307–15. 10.1111/j.1399-0039.2009.01213.x 19317739

[25] Renard C Vaiman M Chiannilkulchai N Cattolico L Robert C Chardon P. Sequence of the pig major histocompatibility region containing the classical class I genes. Immunogenetics (2001) 53:490–500. 10.1007/s002510100348 11685460

[26] Le M Choi H Choi MK Cho H Kim JH Seo HG Development of a simultaneous high resolution typing method for three SLA class II genes, SLA-DQA, SLA-DQB1, and SLA-DRB1 and the analysis of SLA class II haplotypes. Gene (2015) 564:228–32. 10.1016/j.gene.2015.03.049 25824383

[27] Ho CS Martens GW Amoss MS Gomez-Raya L Beattie CW Smith DM. Swine leukocyte antigen (SLA) diversity in Sinclair and Hanford swine. Dev Comp Immunol (2010) 34:250–7. 10.1016/j.dci.2009.09.006 19782700

[28] Smith DM Martens GW Ho CS Asbury JM. DNA sequence based typing of swine leukocyte antigens in Yucatan miniature pigs. Xenotransplantation (2005) 12:481–8. 10.1111/j.1399-3089.2005.00252.x 16202072

[29] Yeom SC Park CG Lee BC Lee WJ. SLA typing using the PCR-SSP method and establishment of the SLA homozygote line in pedigreed SNU miniature pigs. Anim Sci J (2010) 81:158–64. 10.1111/j.1740-0929.2009.00727.x 20438495

[30] Le M Choi H Choi MK Nguyen D Kim JH Seo H Comprehensive and high-resolution typing of swine leukocyte antigen DQA from genomic DNA and determination of 25 new SLA class II haplotypes. Tissue Antigens (2012) 80:528–35. 10.1111/tan.12017 23137324

[31] Essler SE Ertl W Deutsch J Ruetgen BC Groiss S Stadler M Molecular characterization of swine leukocyte antigen gene diversity in purebred Pietrain pigs. Anim Genet (2012) 44:202–5. 10.1111/j.1365-2052.2012.02375.x 22587706

[32] Gimsa U Ho CS Hammer SE. Preferred SLA class I/class II haplotype combinations in German Landrace pigs. Immunogenetics (2017) 69:39–47. 10.1007/s00251-016-0946-6 27484460

[33] Choi NR Seo DW Choi KM Ko NY Kim JH Kim HI Analysis of swine leukocyte antigen haplotypes in Yucatan miniature pigs used as biomedical model animal. Asian Australas J Anim Sci (2016) 29:321–6. 10.5713/ajas.15.0331 26950861 PMC4811781

[34] Ando A Imaeda N Ohshima S Miyamoto A Kaneko N Takasu M Characterization of swine leukocyte antigen alleles and haplotypes on a novel miniature pig line, Microminipig. Anim Genet (2014) 45:791–8. 10.1111/age.12199 25118109

[35] Cho HO Ho CS Lee YJ Cho IC Lee SS Ko MS Establishment of a resource population of SLA haplotype-defined Korean native pigs. Mol Cells (2010) 29:493–9. 10.1007/s10059-010-0061-8 20396963

[36] Zeng R Zeng YZ Molecular cloning and characterization of SLA-DR genes in the 133-family of the Banna mini-pig inbred line. Anim Genet (2005) 36:267–9. 10.1111/j.1365-2052.2005.01277.x 15932417

[37] Zeng R Miao YW Huo JL Pan WR Zeng YZ Molecular cloning and characterization of SLA-DQ cDNA of the Banna mini-pig inbred line family 133. Acta Laboratorium Animalis Scientia Sinica (2005) 13:215–21. Chinese.

[38] Reyes LM Blosser RJ Smith RF Miner AC Paris LL Blankenship RL Characterization of swine leucocyte antigen alleles in a crossbred pig to be used in xenotransplant studies. Tissue Antigens (2014) 84:484–8. 10.1111/tan.12430 25209617

[39] Liu Z Xia J Xin L Wang Z Qian L Wu S Swine leukocyte antigen class II genes (SLA-DRA, SLA-DRB1, SLA-DQA, SLA-DQB1) polymorphism and genotyping in Guizhou minipigs. Genet Mol Res (2015) 14:15256–66. 10.4238/2015.November.30.1 26634489

[40] Gleimer M Parham P. Stress management: MHC class I and class I-like molecules as reporters of cellular stress. Immunity (2003) 19:469–77. 10.1016/S1074-7613(03)00272-3 14563312

[41] Fan S Wu Y Wang S Wang Z Jiang B Liu Y Structural and biochemical analyses of swine major histocompatibility complex class I complexes and prediction of the epitope map of important influenza A virus strains. J Virol (2016) 90:6625–41. 10.1128/jvi.00119-16 27170754 PMC4944273

[42] Wallny HJ Avila D Hunt LG Powell TJ Riegert P Salomonsen J Peptide motifs of the single dominantly expressed class I molecule explain the striking MHC-determined response to Rous sarcoma virus in chickens. Proc Natl Acad Sci U S A (2006) 103:1434–9. 10.1073/pnas.0507386103 16432226 PMC1360531

