# Supplementary material for: Swine Leukocyte Antigen Diversity in Canadian Specific Pathogen-Free Yorkshire and Landrace Pigs
Source: Front Immunol. 2017 Mar 15;8:282. doi: 10.3389/fimmu.2017.00282 (PMC5350106; doi:10.3389/fimmu.2017.00282)
Supplement: Table S2 — Specificities of sequence-specific PCR primers for rapid swine leukocyte antigen (SLA) typing of Canadian specific pathogen-free (SPF) Yorkshire and Landrace pigs. [file Table_2.DOCX]

**Supplementary Table S2** **Specificities of sequence-specific PCR primers for rapid SLA-typing of Canadian SPF Yorkshire and Landrace pigs.**

| Lane | Locus | Allele specificity | GenBank accession number | Pig breed | Product size (bp) | Forward and reverse primer sequence (5′→3′) | Primer position ^a^ |
| --- | --- | --- | --- | --- | --- | --- | --- |
| 1 | Negative control | *ACTA1* |  |  | 516 ^b^ | CGCCATGTGTGACGAAGACGAGACC  CACGTACATGGCGGGCACGTTGAAG | +21  +384 |
| 2 | *SLA-1* | 01:01 | KU754544 | Yorkshire | 209 | GCGAGGCCGGGTCTCACACCTA  CCTCCTACGCTCCGCCACATT | +347  +514 |
| 3 |  | 04:01:01 | KU754545 | Yorkshire, Landrace | 181 | GCCCGACCGCGGGGACTCT  GTAAGTCTGTGCGGTTTCCTTGACA | +123  +261 |
| 4 |  | 07:02 | KU754546 | Landrace | 220 | GCCGGGTCTCACACCATCCAGAT  GGCCCTGCAGGTAGCTCCTCAAT | +353  +528 |
| 5 |  | 08:01  08:07  08:08  08:05 | KU754547  KU953375  KU754548  KU754549 | Landrace  Yorkshire  Yorkshire  Landrace | 141 | CGGGGACTCCCGCTTCTTCATT  GTCTCCCGATCCCAATACTCCG | +135  +233 |
| 6 |  | 09:01 ^c^ |  |  | 193 | CCACTCCCTGAGCTATTTCTT  GATCTGTGTCTCCCGATCCCAATAG | +89  +237 |
| 7 |  | 11:03 | KU754550 | Yorkshire | 183 | GTGTCCCGGCCCGACC  CTGTGCGCTGCCCATGACAC | +112  +260 |
| 8 |  | 12:01 | KU754551 | Yorkshire, Landrace | 186 | GGACTCCCGCTTCATCGAA  GTCTTCAGGTCCACTCGGTTAA | +135  +281 |
| 9 |  | 13:01 | KU754552 | Yorkshire, Landrace | 217 | GTCTCACACCCTCCAGAGCATGTTT  CAGTCCCTGCAGGTAGCTCCTCCTA | +360  +528 |
| 10 |  | 14:01 | KU754553 | Landrace | 500 ^b^ | AGCAGACGCGGAATGTCAT  CCACATCGGCCTCCTCCTG | +266  +502 |
| 11 |  | 07:04 | KU754554 | Yorkshire | 473 ^b^ | GGACTCCCGCTTCATCGCC  AGCCGTACATGTTCTGGAA | +135  +346 |
| 12 |  | 07:03 | KU754555 | Yorkshire | 193 | GGTACAGWCAGTAYGSCTACGACA  TTCCCCATCTCCAGGTATCTGC | +421  +569 |
| 13 |  | 08:12 | KU754556 | Yorkshire | 205 | CGGGGACTCCCGCTTCTTCATT  GTAGTAGCCGAGAGCTGTGCG | +135  +298 |
| 14 |  | 15:01 | KU754557 | Yorkshire | 194 | ACTCCCTGAGCTATTTCTC  GACTTTCCGCGTCTCCTCAG | +89  +245 |
| 15 |  | 08:11 | KU754558 | Yorkshire, Landrace | 150 | CGGGGACTCCCGCTTCTTCATT  16GCGTTCCGCGTGTTCCTATCC | +135  +243 |
| 16 | *SLA-3* | 01:01 | KU754574 | Yorkshire | 174 | CC17TCCGCGGGTACAGTCAGTTT  CACACCACACACGCGCCCTC | +411  +544 |
| 17 |  | 03:06 | KU953377 | Yorkshire | 534 ^b^ | GCGGGAAGCCCCGTTTCATCT  CGTCGTAGGCGTACTGATG | +133  +403 |
| 18 |  | 04:01  04:02  04:04 | KU754575  KU754576  KU754583 | Yorkshire, Landrace  Landrace  Landrace | 192 | GGAAGCCCCGTTTCATCGAA  GCAGGTTTTTCAGGTTCACTCGGA | +135  +284 |
| 19 |  | 04:02 |  |  | 215 | CGACCGCGGGAAGCCCCGT  GGTTGTAGTAGCCGCGCAGC | +126  +303 |
| 20 |  | 05:02  05:03:02 ^d^ | KU754577  KU754582 | Yorkshire, Landrace  Yorkshire | 114 | GTGGACGACACGCAGTTCGTGT  GTCTCCCGATCCCAATACTCCTGA | +166  +231 |
| 21 |  | 06:01 | KU754578 | Yorkshire, Landrace | 153 | GCGACGTGGGGCCAGACT  CATCGGCCGCCTCCCA | +382  +502 |
| 22 |  | 07:01:01  07:01:02 | KU754579  KU754580 | Landrace  Landrace | 153 | CGACCGCGGGAAGCCCCGT  TCCTCATCCCAATACTCCTGCCA | +126  +238 |
| 23 |  | 07:01:02 |  |  | 188 | GCGACGTGGGGCCAGACT  CGCGCCCTCCAGGTAGCTT | +382  +534 |
| 24 |  | 05:06  05:02  05:03:02 ^d^ | KU754581 | Yorkshire | 139 | CGTGGACGACACGCAGTTCGTGT  AGTCTGTGCGTTGTCCTTGCTGA | +166  +260 |
| 25 | *SLA-2* | 01:01 | KU754559 | Yorkshire | 256 | TCCCCACTCCCTGAGCTATTTCTC  GTTGTAGTAGCCGCGCAGGGTC | +98  +309 |
| 26 |  | 04:01  04:02:01 | KU754560  KU754561 | Yorkshire  Landrace | 316 ^b^ | CCGAGGGAACCTGCGCACAGC  CCCACGTCGCAGCCGTACATGA | +314  +362 |
| 27 |  | 05:02  05:04  05:03  05:05 | KU754562  KU754563  KU754572  KU754569 | Landrace  Landrace  Landrace  Yorkshire | 127 | CGGGCGCCGTGGATAGAGA  CCACGCTCTGGTTGTAGTAGCCAAG | +232  +316 |
| 28 |  | 09:03  09:05 | KU953376  KU754567 | Yorkshire  Yorkshire | 181 | CGTGGGACCAGACGGGCTCT  GCCCTGCAGGTAGCTCCTCCAG | +397  +537 |
| 29 |  | 10:01  10:04  10:05  10:06 | KU754564  KU754565  KU754566  KU754570 | Yorkshire, Landrace  Landrace  Yorkshire  Yorkshire, Landrace | 116 | GCCTCGACACAGAATCTCCGCA  CCCGCACTCACCCGCCTGA | -11  +66 |
| 30 |  | 16:03 | KU754568 | Yorkshire | 175 | CGGGGACTCCCGCTTCCTCA  TCTGTAAATCTGTGCGGTTTCCTTGTAA | +142  +270 |
| 31 |  | 06:02:01 | KU754571 | Landrace | 127 | CGCCCCGAATCCGAGGAAA  CAGGGTGTTCAGGCCCACTCGGTA | +207  +292 |
| 32 |  | 02:02 | KU754573 | Landrace | 139 | CCTCCGCGGGTACAGTCAGTTT  CTGCTCCGCCACATTGGCT | +420  +519 |
| 33 | *DRB1* | 02:01:01 | KU754592 | Yorkshire | 115 | GCATTTCTTGCACCTGTTGAAATTC  GTCGCTGTCGAAGCGCAGGAA | +126  +196 |
| 34 |  | 04:02 | KU754593 | Yorkshire | 206 | CGCATTTCTTGTTTCTGSGGAAGG  CGCCCGCTTCTGCTCCAT | +124  +289 |
| 35 |  | 05:01 | KU754594 | Landrace | 172 | GGACGGAGCGGGTGCT  GTGTCCACTGAGGCCCGTGAGTC | +161  +295 |
| 36 |  | 06:01  06:02  06:07 ^d^ | KU754595  KU754596  KU754601 | Landrace  Landrace  Yorkshire | 122 | GGGTGAGGTATCTGCTGAAGTACTTG  GCTGTTCCAGTACTTGGCGTCT | +180  +255 |
| 37 |  | 07:01 | KU754597 | Yorkshire | 133 | GGACCGAGCGGGTGAGGTTCA  TGGCTGTTCCAGTACTTGGCTGAA | +166  +255 |
| 38 |  | 08:01 | KU754598 | Landrace | 108 | CCCACCGCATTTCTTGTACCTGT  CGCACGTACTCCTCTCCGTTATAGTAC | +118  +177 |
| 39 |  | 09:01:01 | KU754599 | Yorkshire | 157 | TTCTTCAATGGGACCGAGCA  CCAGGAGGTCCTTCTGGCTGTTAT | +155  +269 |
| 40 |  | 10:01 | KU754600 | Yorkshire, Landrace | 135 | CCCCACCGCATTTCTTCTTTATGGA  GGTACTCGCCCACGTCGCTA | +119  +210 |
| 41 | *DQB1* | 02:01  02:02  02:12 ^d^  02:03 | KU754584  KU754585  KU754590  KU754591 | Yorkshire, Landrace  Yorkshire  Yorkshire  Landrace | 146 | GGGGCGTGGCCAGGTGGG  GGCCCGCTTCTGCTCCAGGAC | +187  +295 |
| 42 |  | 04:01:01 | KU754586 | Yorkshire | 197 | CAGCGGGTGTGGAGCGTGGA  TCCTCTATCTGGTAGTTGTGTTTGCACACA | +179  +327 |
| 43 |  | 06:01 | KU754587 | Yorkshire, Landrace | 204 | ACGCAGCGGGTGCGGCA  GCCTTCCTCTATCTGGTAGTTGTGTTTGC | +173  +332 |
| 44 |  | 07:01:01 | KU754588 | Landrace | 154 | CACGTGCGCTTCGACAGCA  CTTCCTCTATCTGGTAGTTGTGTTTGCACACA | +223  +327 |
| 45 |  | 09:01 | KU754589 | Yorkshire | 146 | TGCGGCTCGTGACCAGATT  CCGCGTCTGCTCCAGGAT | +185  +295 |

*^a^ Nucleotide position of SLA genes corresponding to the 3′ terminus of the primer.*

*^b^ Product spans intron.*

*^c^ SLA-1*09:01 was detected by PCR-SSP but missed by mRNA SBT.*

*^d^ Unconfirmed allele by the ISAG SLA Nomenclature Committee.*
